# Editorial: Insights in genome editing in animals 2023/2024

**DOI:** 10.3389/fgeed.2025.1556425

**Published:** 2025-01-23

**Authors:** Gӧtz Laible, Mark Cigan

**Affiliations:** ^1^ Animal Biotechnology, Ruakura Research Centre, AgResearch, Hamilton, New Zealand; ^2^ Department of Molecular Medicine and Pathology, School of Medical Sciences, University of Auckland, Auckland, New Zealand; ^3^ Cobb-Vantress, Siloam Springs, AR, United States

**Keywords:** genome editing, regulatory, Livestock, applications, PRRSV resistance, large animal models, surrogate sire technology

The development of programmable nucleases for site-specific, precise editing of animal genomes has enabled the introduction of an almost unlimited range of genetic modifications, previously reserved for small animal models such as the mouse. This has opened opportunities for applications in commercially relevant animal species to accurately study gene function, develop superior models of human diseases and improve food producing animals. While genome edited plants are well on their path to make an impact with commercialized varieties (Pixley et al., 2022), animals have so far been held back by still developing regulations for genome edited animals. This insight Research Topic comprises six articles ranging from an overview of applications on the horizon, a review highlighting the impact of genome editing on large animal research, two original studies and a review about the regulatory challenges faced by genome edited animals.

Miklau et al. have used published information to identify current developments of genome edited animals and microorganisms. The review provides a forecast for genome edited animals expected to be soon interacting with the environment. Considered to be relevant in this context were 70 and 57 studies involving terrestrial animals and fish, respectively. The main applications on the horizon for these two categories were aimed at disease resistance and reproduction. The study provides valuable information for the assessment of environmental risks that will strongly depend on species and traits.

The review by Fischer and Schnieke illustrates how technological advancements from pronuclear microinjection to the cost-effective, highly flexible CRISPR/Cas9 technology has changed the ability to develop large animal models for agricultural and biomedical applications. Homozygous knockouts (KOs) of multiple genes can be achieved with high efficiency. They also describe the rapid improvement of genome editors and novel, more efficient strategies for their delivery. It is balanced against shortcomings of the technology with the potential for off-target events and mosaicism. Moreover, the improved technical abilities of genome editing are associated with a reduction of the number of animals used for the generation of complex animal models compared to previous, older approaches. Although reduction of experimental animals is a major aim in the 3R guiding principles for improving animal welfare, this benefit in the application of genome editing is often overlooked.

Han et al. investigated the role of non-coding DNA sequence elements involved in gene regulation in chickens, focusing on the discovery of enhancer and promoter regions using CRISPR activation technology. Often these non-coding regulatory regions harbor sequence variants linked to Mendelian disorders and have been identified as having a role in disease susceptibility and phenotypic traits. Using dead Cas9 fused to activation domains to manipulate HBBA, IRF7, and PPARG genes in chicken DF-1 cells, the researchers were able to identify and discriminate functional enhancers from promoters and validate enhancer regions identified using epigenomic data from the FAANG project. This research provides a foundation to expand the understanding of enhancer functions in the chicken genome and discover how sequence variation in non-coding regions contribute to the advancement of poultry breeding programs at the DNA level.

Mueller et al. investigates the function of NANOS3 in bovine germline development by generating KO cattle using the CRISPR/Cas9 system as a surrogate system to accelerate trait improvement in cattle. The NANOS gene family plays a vital role in germ cell development across species for the germline of both male and female. As demonstrated in pigs (Kogasaka et al., 2022), male and female NANOS3 KO animals show a complete loss of germ cells, though gonadal development remains unaffected. Using a dual gRNA/Cas9 approach, NANOS3 was efficiently disrupted in bovine embryos. In these KO cattle, primordial germ cells were completely eliminated in fetal testes while seminiferous tubule development was unaffected. At sexual maturity, the NANOS3 KO bull exhibited normal reproductive traits, while the heifer showed compromised ovarian development, highlighting a greater impact on female germline development. This work demonstrates that NANOS3 is essential for both male and female germline development in cattle and highlights the potential use of NANOS3 KO cattle as an advanced breeding technology.

The article by Nesbitt et al. explores the suitability of a genome edited line of pigs for its seamless integration into commercial production systems. The homozygous deletion of exon 7 in the CD163 gene renders these pigs resistant to infections by the porcine reproductive and respiratory syndrome virus. While the disease-resistance phenotype has been proven, the study investigates whether the genome edited genotype is stable or might have any negative impacts on important production traits. The data reveal that the resistance phenotype was stable over generations and no changes were observed in the edited pigs’ general health and wellbeing, fertility, production characteristics, meat composition and meat quality. This provides evidence that these edited pigs with their improved welfare and economic credential are ready to be commercialized following regulatory approval.

As mentioned above, genome editing holds significant potential for advancing animal agriculture by enabling rapid trait incorporation, such as growth enhancement, disease resistance, and novel phenotypes, into selectively bred animals without relying on traditional crossbreeding or backcrossing methods. In the review by Wray-Cahen et al., challenges of genetically modified organisms (GMOs) entering conventional production are reviewed in the context of international regulatory frameworks. While the advances in the technology have historically outstripped regulatory frameworks globally, new regulatory guidelines, pioneered by Argentina, are beginning to change the landscape allowing genome-edited organisms, which could have been developed using conventional methods, to be regulated similarly to non-GMO organisms. Wray-Cahen et al. stresses that regulatory policies should focus on using genome editing technologies as tools within existing breeding programs to incorporate new traits, rather than only creating entirely new products. This shift would be expected to improve the prospects for biotech animals to enter commercial production necessary to combat diseases not achievable by classical breeding methods.

The article collection highlights the step change in the targeted modifications of animal genomes by genome editing and with it the necessary update of risk assessment and regulations. Importantly, it shows that genome edited animals are getting closer to realize their benefits towards greater animal welfare, sustainable food production and human health.
